# Characterization of Novel Luteoviruses in Canadian Highbush Blueberries Using High-Throughput Sequencing

**DOI:** 10.3390/v17101286

**Published:** 2025-09-23

**Authors:** Sachithrani Kannangara, Adam Gilewski, Juan Rodriguez Lopez, Gertruida de Villiers, Meghan Ellis, Peter Ellis, Eric Gerbrandt, Jim Mattsson

**Affiliations:** 1 Department of Biological Sciences, Simon Fraser University, 8888 University Dr, Burnaby, BC V5A 1S6, Canada; sachithrani_kannangara@sfu.ca (S.K.); juan_rodriguez@sfu.ca (J.R.L.); 2 Phyto Diagnostics Company Ltd., 9381 Ardmore Dr, North Saanich, BC V8L 5G4, Canadaellisp@phytodiagnostics.com (P.E.); 3 British Columbia Blueberry Council, 275-32160 S Fraser Way, Abbotsford, BC V2T 1W5, Canada; research@bcberries.ca

**Keywords:** luteovirus, blueberry disease diagnostics, high-throughput sequencing, virus detection, virus genome

## Abstract

The Fraser Valley of British Columbia, Canada is among the top ten blueberry producing regions globally. Viral diseases are established in the region and significantly reduce average yields. While testing for two viruses is routine, characterization of all the viruses present in the region is incomplete. We used high-throughput sequencing to obtain an unbiased overview of RNA viruses present in 97 plants collected across the region. In addition to known viruses, we identified four luteoviruses previously unidentified in the region. Two of them matched the blueberry virus L (BlVL) and blueberry virus M (BlVM). recently found in the USA, while the third constitutes a new major variant of BlVM (BlVM-2), and the fourth a new luteovirus, which we named blueberry virus N (BlVN). The genome sequences were ~5 kbp long and contained four open-reading frames similar to other luteoviruses. PCR screening revealed that these luteoviruses are widespread in the region, and that plants typically harbour more than one of these luteoviruses. While luteoviruses are typically vectored by aphids, they were also present in nursery stock, indicating that spread also occurs via vegetative propagation.

## 1. Introduction

Canada cultivates more highbush blueberry (*Vaccinium corymbosum* L.) than any other fruit commodity [1]. The vast majority (95%) is produced in the Fraser Valley of British Columbia (BC), where blueberries and other berries have been grown commercially for many decades [2]. Viral diseases severely impact production in the region. A number of viruses are associated with highbush blueberry diseases, including blueberry fruit drop virus (BFDaV), blueberry latent virus (BlLV), blueberry leaf mottle virus (BLMoV), blueberry mosaic-associated virus (BlMaV), blueberry red ringspot virus (BRRV), blueberry scorch virus (BlScV) and closely related blueberry virus S (BlVS), blueberry shock virus (BlShV), blueberry shoestring virus (BlSSV), blueberry virus A (BVA), peach rosette mosaic virus (PRMV), tobacco ringspot virus (TRSV), and tomato ringspot virus (ToRSV) [3,4,5,6,7]. Among them, BlScV (genus *Carlavirus*) and BlShV (genus *Ilarvirus*) viruses are the most prevalent in BC, causing widespread disease and many millions of dollars each year in lost revenue. They are also considered a high-risk pathogen in Northwest America [8,9,10]. BFDaV, BlLV, BlMaV and ToRSV are also present in the region, although less common and less damaging [8].

Recently, a high-throughput sequencing (HTS) sequencing approach was used to screen blueberry plants at several locations in the USA for unknown viruses. These studies revealed two novel viruses in the *Luteovirus* genus, which were named blueberry virus L (BlVL) [4] and blueberry virus M (BlVM) [11]. BlVL has also been detected in Japan [12]. The pathogenicity of these novel blueberry viruses is still unknown [4,11]. Luteoviruses are transmitted by aphids, which harbour the virus particles in a persistent, circulative and non-propagative manner [13]. Aphids transmit luteoviruses via the stylet when feeding on phloem sap [14]. In plants, luteovirus particles are found to be restricted to the phloem and companion cells and can be transmitted by aphids again following a short latency period [15]. Luteoviruses belong to the *Tombusviridae* family, which includes well-known plant viruses such as Barley Yellow Dwarf Virus (BYDV) and Potato Leaf Roll Virus (PLRV) [13,16]. Luteoviruses cause disease in a wide range of crop species, including monocot cereal crops, dicot crops such as soybeans, beans, and woody plants such as apples, nectarines, cherries, and roses [13,15,16,17]. Luteovirus infections often lead to symptoms such as yellowing or discoloration of leaves, stunting, and reduction in crop quality and yield. However, symptoms and the severity of the symptoms can vary depending on the specific luteovirus and the host plant [15]. Luteoviruses have been identified by HTS in different crop species, including nectarine stem-pitting associated virus (NSPaV) [18], red clover-associated luteovirus (RCaV) [19] and apple-associated luteovirus (AaLV-1) [20]. The NSPaV-infected nectarine trees are reported to show symptoms like stunting and stem pitting. However, RCaV was reported to co-infect with other viruses, and the symptoms directly caused by this luteovirus are unknown [19]. Further, AsLV-1 infected apple trees showed no symptoms. [20].

Luteoviruses contain a single-stranded positive-sense RNA genome ranging from 5.6 to 6 kb in length. Viruses in this family have an icosahedral non-enveloped capsid that encapsulates the monopartite RNA genome [21]. Most luteovirus genomes encode a replicase protein (P1 and P1-P2 fusion protein), a capsid protein (CP), a CP-readthrough protein, and a movement protein (MP) [13]. The P1-P2 fusion protein has both helicase and RNA-dependent RNA polymerase (RdRP) activities needed for replication of the virus genome and generation of sub-genomic RNAs [4,20]. The C-terminal extension of the P1 protein with the P2 protein is controlled by a ribosomal frameshift mechanism that has been characterized [15,21]. This frameshift is triggered by a conserved heptanucleotide frameshift element adjacent to the stop codon of ORF1 and a conserved downstream element that generates a secondary structure [22]. The third nucleotide of the frameshift element varies in different luteoviruses [4,18,19,20]. ORF3 encodes the virus capsid protein, which also have other functions [15]. ORF3-ORF5 readthrough proteins are known to facilitate the aphid-mediated movement of virus particles. Most but not all luteoviruses have an MP-encoding ORF4 embedded within the ORF3 [17,18,19]. Both BlVL and BlVM contain a ~5 kbp single-stranded RNA genome with a genomic structure similar to the closest relative, the nectarine stem-pitting-associated virus (NSPaV). The genome of both viruses carries ORFs encoding P1 protein (ORF1), P1-P2 fusion protein (ORF1-2), capsid protein (ORF3) and a coat protein readthrough protein (ORF3-5). Only BlVL contains an extra ORF embedded in ORF5 encoding a putative protein P5b. Unlike most luteoviruses, both BlVL and BlVM genomes lack the MP-encoding ORF4 [4,11].

Independently of the studies reported in [4,11] we used HTS to screen 87 diseased and 10 healthy plants from across blueberry farms in the BC Fraser Valley to assess if there are novel viruses present in the region. In addition to expected BlScV and BlShV, we identified four luteovirus-like sequences previously unknown to occur in the Fraser Valley. Three of the luteovirus-like sequences matched BlVL, BlVM and a variant of BlVM, while the fourth constituted a new luteovirus. We named the novel luteovirus as blueberry virus N (BlVN) and the variants of BlVM as blueberry virus M-2 (BlVM-2). Their sequences, genome organization, and spread in the Fraser Valley are described here.

## 2. Materials and Methods

### 2.1. Sample Collection

Blueberry shoots, 20–30 cm in length, were collected from about 200 plants with scorch-like symptoms from commercial fields in the Fraser Valley in the municipalities of Chilliwack, Abbotsford, Langley, Surrey, Delta, and Pitt Meadows between June and July 2020. Shoots were also collected from symptomless plants. A subset of 87 diseased plants and 10 symptomless plants were sampled for RNA-seq. For subsequent PCR screening, diseased leaves were collected by consultants and farmers across the Fraser Valley in 2022 (provided by Phyto Diagnostics Company Limited., Saanich, BC, Canada) and leaves of healthy plants were purchased from local garden stores and tested. Plant leaf samples were stored at −20 °C and later used for RNA extraction.

### 2.2. RNA Extraction from Blueberry Leaves

Total RNA was purified using a modified version of the activated charcoal/CTAB RNA extraction protocol by Rajakani et al., 2013 [23]. Leaf samples, 75–80 mg, were flash-frozen and ground in 2 mL tubes with 2.0 mm Zirconia beads using a bead beater (2400 rpm for 1 min, Mini-Bead Beater-96, BioSpecs products, Bartlesville, OK, USA). Immediately thereafter, 800 µL of pre-heated (65 °C) extraction buffer (0.5% activated charcoal, 1.5% PVPP, 1.5% β-mercaptoethanol) and buffer (2% *w*/*v* CTAB, 0.2 M Tris-Cl pH 8.0, 50 mM EDTA, 2.5 M NaCl) was added to each tube. Samples were incubated at 65 °C for 15 min after homogenizing. Next, 800 µL of chloroform was added, tubes shaken and centrifuged (25–32 k RCF, for 5–10 min at 4 °C), followed by collection of the water phase. This step was repeated to increase the purity of RNA. Then 1/4 volume of 10 M LiCl was added, samples mixed by inversion, and left at 4 °C overnight to precipitate the RNA. The next day, RNA was collected by centrifugation at 25–32 k RCF for 30 min. After decanting, the pellet was washed with 80% ethanol. This step was repeated to ensure the removal of all LiCl. After decanting, tubes were centrifuged to collect the remaining ethanol, which was carefully removed by pipette, and dried for 5 min. RNA pellets were resuspended in 11 µL of RNase-free water. Next, residual genomic DNA was removed using the RapidOut DNA Removal Kit (Thermofisher Scientific, Mississauga, ON, Canada, Cat. No. K2981) according to the manufacturer’s instructions. The concentration and quality of RNA were checked by spectrophotometry and denaturing gel electrophoresis [24].

### 2.3. RNA-Seq Library Preparation and Data Analysis

Before library preparation, ribosomal RNA was removed using the QIAseq FastSelect –rRNA Plant Kit (QIAGEN, Mississauga, ON, Canada, Cat. No. 334315). RNA-seq libraries were constructed using the NEBNext Ultra II RNA Library Prep Kit for Illumina (New England Biolabs, Ipswich, MA, USA, Cat. No. E7775L). Each cDNA library was tagged during PCR enrichment (11 cycles) with next generation sequencing (NGS) Unique Dual-Index Primers (Eurofins Genomics, Louisville, KT, USA). The pooled libraries were sequenced on an Illumina NovaSeq 6000 machine using S4 flow cells and the paired-end 150 base read option (The Centre for Applied Genomics, Hospital for Sick Children, Toronto, ON, Canada).

RNA-seq data were analyzed using the following workflow. Paired reads were trimmed to remove sequencing adaptors and low-quality regions using Trimmomatic [25]. The read quality was verified using FastQC Version 0.12.0 (https://www.bioinformatics.babraham.ac.uk, accessed on 19 August 2022), and PCR-duplicated reads were removed before assembly. For each sample, paired reads were separately assembled de novo into contiguous sequences (contigs and scaffolds) using the rnaviralSPades pipeline in the SPades assembler [26]. The assembled scaffolds were filtered by length (>200 bp) and clustered with MMseq2 greedy incremental clustering (clustering mode 2) with a minimum sequence identity of 0.98 and a coverage threshold of 0.5. Only one representative sequence was kept from each cluster. The selected contigs were further filtered according to their read coverage per sample (lengthscaled TPM [Transcripts Per Kilobase Million] ≥10 at least in 2 samples and ≥50 across all samples) that quantified using Salmon 1.10.2 package [27]. MMseq2 was used for taxonomy assignment of the sorted assemblies. The taxonomy reference database was created with the NCBI nr database (https://ftp.ncbi.nlm.nih.gov/pub/taxonomy/taxdump.tar.gz, 5 June 2023). Taxonomic labels were assigned by 2bLCA protocol (lca mode 4) of MMseqs2. Contigs of potential pathogen genomes were further analyzed using map to reference function Geneious prime^®^ 2023.2.1 software. We obtained a consensus sequence for each positive sample using this function. Only consensus sequences with high read coverage (>80%) were considered for further analysis.

### 2.4. Genome Structure and Phylogenetic Analysis

The ORF prediction tool in Geneious Prime was used to annotate the open reading frames (ORFs) in blueberry luteovirus consensus sequences. The ORFs were further confirmed by multiple sequence alignment with reference sequences of other luteoviruses available in GenBank and using NCBI BLAST + 2.14.1 tool to search against the nucleotide (nr) database. Multiple sequence alignment (MSA) was generated with ClustalW with nine related luteoviruses, BlVL and BlVM reported from the USA. The best DNA/protein model was predicted using the MSA, and then a phylogenetic analysis was conducted using the maximum likelihood (ML) method with the general time reversible model (GTR). Evolutionary analyses were conducted in MEGA12 [28].

### 2.5. Validation by RT-PCR and Amplicon Sequencing

The presence of BlVN and BlVM/M-2 was confirmed using PCR. The alignment of consensus sequences (Clustal Omega) was used to generate new primers targeting conserved regions in coat protein coding sequences using Geneious prime. Primers were validated by testing samples collected from different locations in the Fraser Valley. RNA from leaf material was extracted as described above. Then, cDNA synthesis was carried out with gene-specific primers according to the manufacturer’s instructions for the OneScript Hot cDNA Synthesis Kit (Applied Biological Materials Inc., Richmond, BC, Canada, Cat. No. G594). PCR was conducted with blueberry luteovirus-specific primers (Table 1). Fifteen µL reaction mixes contained Taq buffer (1.5 mM MgCl2, 50 mM KCl, 10 mM Tris. HCl, pH 8.0), 0.2 mM of each dNTP, 0.4 µM of each primer, 0.75 U Taq DNA polymerase (Applied Biological Materials Inc, Cat. No. G009) and 1 µL of template cDNA for PCR cycling with initial denaturation at 94 °C for 3 min; 30 cycles of 30 s at 94 °C, 30 s at 57 °C and at 1 min at 72 °C; and the final extension at 72 °C for 5 min. The primer sequences and annealing temperatures are detailed in Table 1. The PCR products were visualized on 1.5% agarose gels.

The coat protein regions of the viral sequences were further sequenced to determine the sequence diversity of BlVN and BlVM/M-2 (Table 1). The primers were designed to amplify the complete coat protein coding sequence (CDS) of blueberry luteoviruses from 32 samples. Those amplicons were purified (QIAquick PCR Purification kit, QIAGEN, Mississauga, ON, Canada, Cat. No. 28106) and sent to the Sequencing and Bioinformatics Consortium, University of British Columbia (Vancouver, BC, Canada) for Sanger sequencing [29]. Sequencing results were analyzed by multiple alignment with the ClustalW Algorithm (Geneious Prime). Further, phylogenetic analysis of the nucleotide and amino acid sequence variations was performed by the ML method with the GTR, and Jones-Taylor-Thornton (JTT) models, respectively with 1000 bootstrap replicates. Evolutionary analyses were conducted in MEGA12 [28].

### 2.6. Confirmation of Genome Sequence of BlVN and BlVM-2

Near full-length N and M-2 blueberry luteoviruses were sequenced using Oxford Nanopore technology. A two-step RT-PCR protocol was used to amplify 5 kbp fragments from each virus. First, cDNA synthesis was carried out using Superscript IV Reverse Transcriptase (ThermoFisher Scientific, Mississauga, ON, Canada, Cat No. 18090010) according to the manufacturer’s protocol with specific primers designed to amplify BlVN and BlVM genome sequences. Nested PCR was conducted with the LongAmp Taq PCR Kit (New England Biolabs, Cat. No. E5200S) and designed primers (Table S1). The lengths of amplicons were assessed by agarose (0.8%) gel electrophoresis. Amplicons with correct size were purified and sequenced by Oxford Nanopore sequencing utilizing v14 library prep chemistry and primer-free sequencing using the R10.4.1 flow cells (Eurofins Genomics, Louisville, KT, USA). The near full-length genomes of selected isolates were analyzed by pairwise sequence alignment with ClustalW and a sequence identity matrix generated by the Sequence Demarcation Tool (SDT)—version 1.2 [30]. The genome ends of BlVN were amplified using 5′/3′ RACE Kit, 2nd Generation (Roche Diagnostics, Mannheim, Baden-Württemberg, Germany, Cat No 03353621001), according to the manufacturer’s instructions. The 3′ end was polyadenylated using an RNA polyA Tailing Kit (Molecular Cloning Laboratories, San Francisco, CA, USA) before 3′ RACE amplification. Amplified products were sequenced using Illumina amplicon sequencing (Amplicon-EZ, Azenta Life Sciences, South Plainfield, NJ, USA). The genome sequences of BlVN and BlVM-2 were aligned, and primers were designed to differentiate the two viruses (Table 2).

## 3. Results

### 3.1. Novel Blueberry Luteovirus and Phylogeny

The taxonomy results (MMSeq2) for virus-like de novo assembled scaffolds revealed the presence of luteovirus-like contigs (14% of virus contigs) in the blueberry RNA-seq data. Together with the more prevalent blueberry scorch virus (BlScV, genus *Carlavirus*) (35%) and blueberry shock virus (BlShV, genus *ilarvirus*) (7%) assemblies. Moreover, we found contigs belong to other blueberry viruses at low levels, including blueberry latent virus (BBLV, genus *Amalgavirus*) (2%), and blueberry mosaic-associated virus (BlMaV, genus *Ophiovirus*) (<1). The luteovirus-like contigs were further analyzed by mapping RNAseq reads of each sample to generate consensus sequences per sample. We identified two distinctive groups/clades of luteovirus consensus sequences using Multiple sequence alignment (MSA) based on indels and SNPs of both nucleotide and amino acid sequences. The first clade of luteovirus sequences had 99.5% nucleotide sequence identity (N seq ID) to the partial genome sequence of Riboviria species isolate ctZ591 (BK035257), which was found in a metagenomic study of human gut virome [31]. We found that this group had 64.2% nucleotide sequence similarity to BlVL (OQ686746.1) genome and 84.3% nucleotide sequence similarity to BlVM (OR051501.1), which were first detected in blueberries across the USA [4]. Consistent with the letter codes of new blueberry viruses, we named this novel luteovirus sequence as blueberry virus N (BlVN). The MSA of each translated ORFs of BlVN against its closest relatives BlVL and BlVM indicated that BlVN is a separate species, according to the International Committee on Taxonomy of Viruses (ICTV) species demarcation criteria for *Luteovirus*, as any luteovirus with more than 10% difference between the amino acid sequence of any gene product belongs to a separate species in the *Luteovirus* genus [32]. Pairwise sequence comparison of translated ORFs of BlVN and BlVM/M-2 showed a difference greater than 10% between amino acid seq ID, except in P1-P2 fusion protein (9.3%), separating them as two distinct viruses in the *Luteovirus* genus (Table 3). Further, the MSA of both nucleotide and amino acid sequences of BlVN and BlVM/M-2 with BlVL showed a greater difference between seq IDs (>20%).

**Figure 1:**
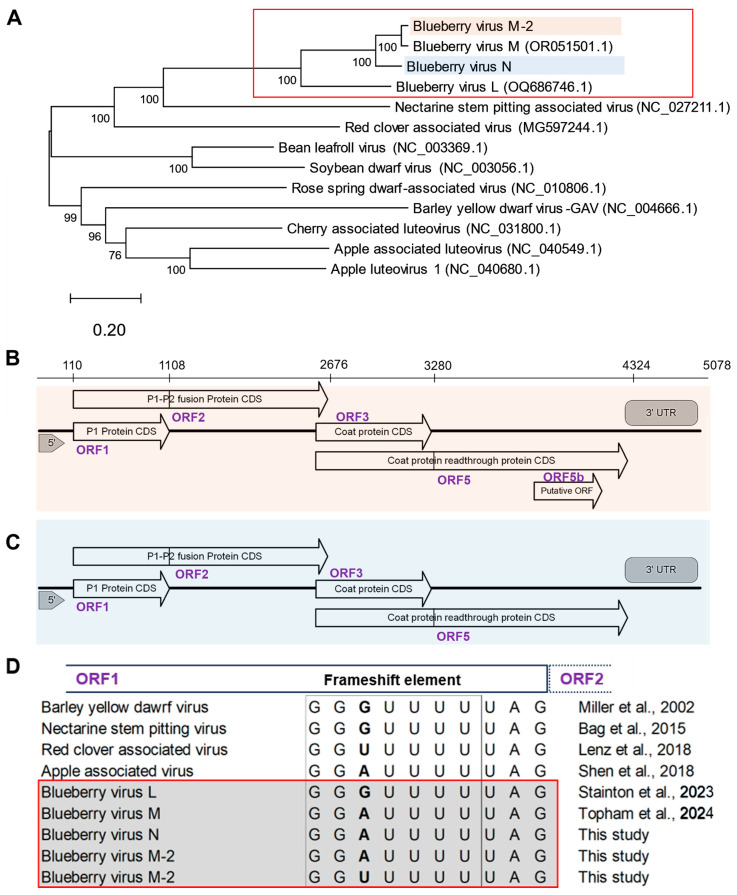
Blueberry luteovirus genome phylogeny and organization. (**A**) The evolutionary relationship between novel blueberry luteoviruses and other related luteoviruses, inferred by the maximum likelihood method and general time reversible model with 1000 bootstrap replicates. The tree is drawn to scale, with branch lengths measured in the number of substitutions per site and the percentage consensus support indicated at each node. Analysis conducted in MEGA12. The related luteovirus are retrieved from NCBI GenBank and accession numbers are indicated within brackets. (**B**) A diagram of predicted genome organization with putative open reading frames of blueberry virus N and (**C**) blueberry virus M-2; ORF1 encoding a P1 protein, ORF1-2 encoding a P1-P2 fusion protein, ORF3 encoding a capsid protein and ORF3-5 encoding coat protein readthrough protein. (using ORFfinder in Geneious Prime and NCBI BLAST). (**D**) Comparison of the heptanucleotide ribosomal frameshift element in ORF1 of blueberry virus L, N, M, and M-2 with other related luteoviruses [4,11,18,19,20,21].

The second group of luteovirus sequences was more closely related to BlVM [11]; thus, we identified it as a sequence variant of blueberry virus M, BlVM-2. BlVN and BlVM-2 genomes have 84.6% N seq ID. Moreover, the BlVM-2 genome has 65.2% and 96.6% N seq ID to the BlVN and BlVM genomes, respectively. The ML tree of BlVN and BlVM-2 with related luteoviruses showed clustering of BlVN, BlVM-2 with BlVM, and BlVL, all being well separated from other related luteoviruses (Figure 1A). BlVN and BlVM-2 were closely related to other known luteoviruses, such as nectarine stem pitting-associated virus (49.5% and 48.7% of N seq ID, respectively), red clover-associated virus (48.2% and 48.4% of N seq ID, respectively), and cherry-associated luteovirus (45.6% and 45.8% of N seq ID, respectively). Moreover, we found de novo assembled contigs that were 4998 to 5048 nucleotides (nts) long and closely related to BlVL, showing the presence of BlVL in the Fraser Valley region, BC. These BlVL-like contigs showed 93% pairwise N seq ID with the USA BlVL isolate OQ686746.1 (Table S2).

### 3.2. Genome Structure of Blueberry Luteoviruses

The de novo assembled BlVN and BlVM-2 genome sequences contained 5078 and 4968 base pairs, respectively, with four common putative open reading frames (ORFs) (Figure 1B,C). ORF1 is located between nts 110-1108 and nts 92-1090 of BlVN and BlVM-2 genomes, respectively, with high sequence similarity to the P1 protein of the *Luteovirus* genus known to have helicase activity [15]. In these ORFs we found the expected conserved GG(**A/U**)UUUU heptanucleotide frameshift element immediately before the stop codon, which is needed to facilitate a -1 ribosomal frameshift to encode ORF2 (between nts 110-2676 in BlVN and nts 92-2658 in BlVM-2) as a P1-P2 fusion protein (variable base in bold font) [21]. All the BlVN isolates contained ‘GG**A**UUUU’ in the frameshift element (Figure 1D). BlVM-2 isolates also contained ‘GG**A**UUUU’ except for three isolates (LU142, LU315, and LU387), which contained ‘GG**U**UUUU’. Both BlVN and BlVM-2 sequences contained the second conserved region vital for frameshifting, i.e., ‘CCCGUUUUCUAUUUUGGG’ 30 nt downstream to heptanucleotide sequence, which is predicted to form a loop structure [15,18]. We confirmed the prediction using the RNA fold function in Geneious prime. Some isolates of BlVM contained one SNP in the fourth nucleotide position of the conserved region, i.e., ‘CCC(**C/G**)UUUUCUAUUUUGGG’.

We compared the P1-P2 amino acid sequences of BlVN and BlVM-2 with BlVL, BlVM and other related luteoviruses to identify conserved motifs with known helicase and RdRp activity [33,34]. Three similar helicase motifs (P1 motif III, P2 motif IV, and P2 motif VI) were found in the P1-P2 ORFs in BlVL, BlVN, and BlVM/M-2 (Figure S1a) [4,33]. Further, we identified eight previously reported positive-strand RNA virus RdRp motifs in BlVL, BlVN, and BlVM/M-2 [34]. Among them, motifs I, VI, VII, and VIII were identical in all BlVL, BlVN and BlVM/M-2, while motifs II, III, IV and V were variable in BlVN and BlVM/M-2 compared to BlVL [4]. RdRp motif II was present as PRLICTKRYNVE**L**GRRLKFN, motif IV present as **R**SPVAIGVDASRF and motif V was present as **K**GHRMSGDINTSMGNKLVMCGMMHN in both BlVN and BlVM/M-2. Further, RdRp motif III was present in BlVN and BlVM/M-2 as VLSGYDNFNVGR**I**I**A**KKWR and VLSGYDNFNVGR**L**I**E**KKWR, respectively (variable amino acids are highlighted in bold) (Figure S1b).

ORF3 of BlVN (2645–3272 nt) and BlVM/M-2 (2635–3262 nt) encoded a viral coat protein (CP), with 208 amino acids. The stop codon of ORF3 was an amber stop codon (UAG), consistent with other luteoviruses [4,18,20] (Figure 1B). Further, we found a potential ORF, of unknown function in BlVN, embedded within ORF5 (ORF5b), spanning from 3733 to 4080 (102 amino acids long). However, we found no evidence of a potential ORF embedded within ORF5 of the BlVM-2 genome (Figure 1C). Phylogenetic trees of ORFs from BlVN, BlVL, and BlVM/M-2 showed similar topology as Figure 1A, with ORF1-2 and ORF3 trees shown in Figure S2.

### 3.3. Prevalence of Blueberry Luteoviruses in the Fraser Valley

The prevalence of blueberry luteoviruses in the Fraser Valley was evaluated by RT-PCR testing of two populations. The first population of 213 symptomatic blueberry plants were tested with LutF2-LutR1 (Table 1, Figure S3a), and results show that 91% were positive for BlVN or BlVM/M-2 (Table 4). Further, we tested samples from 153 plants sent by farmers for virus testing (test population 2) with both LutF2-LutR1 and BlVL primers [4]. Results show that plants typically contain a combination of BlVL, BlVN, and BlVM/M-2 (Table 5). Altogether, more than 80% of diseased blueberry samples tested positive for blueberry luteovirus in every region.

We observed that many symptomatic blueberry plants that tested positive for BlScV or BlShV, were also infected with BlVN and BlVM/M-2. In Population 1, 97.6% of BlScV-infected plants and 98.5% of BlShV-infected plants also tested positive for luteoviruses. Similarly, in Population 2, 95% of plants infected with BlScV and 81% of those infected with BlShV tested positive for luteoviruses. Luteoviruses were found at a similar frequency (87.5%) among plants that tested negative for both BlScV and BlShV. In a cursory screen of limited population sizes, luteoviruses were detected in 50% of apparently healthy plants sampled from commercial fields and 28.7% of plants sampled from commercial nursery stock (Table 4), showing the presence of luteoviruses also in these two categories of plants.

### 3.4. BlVN and BlVM/M-2 Sequence Diversity

#### 3.4.1. Amplicon Sequencing of Virus Coat Protein

**Figure 2:**
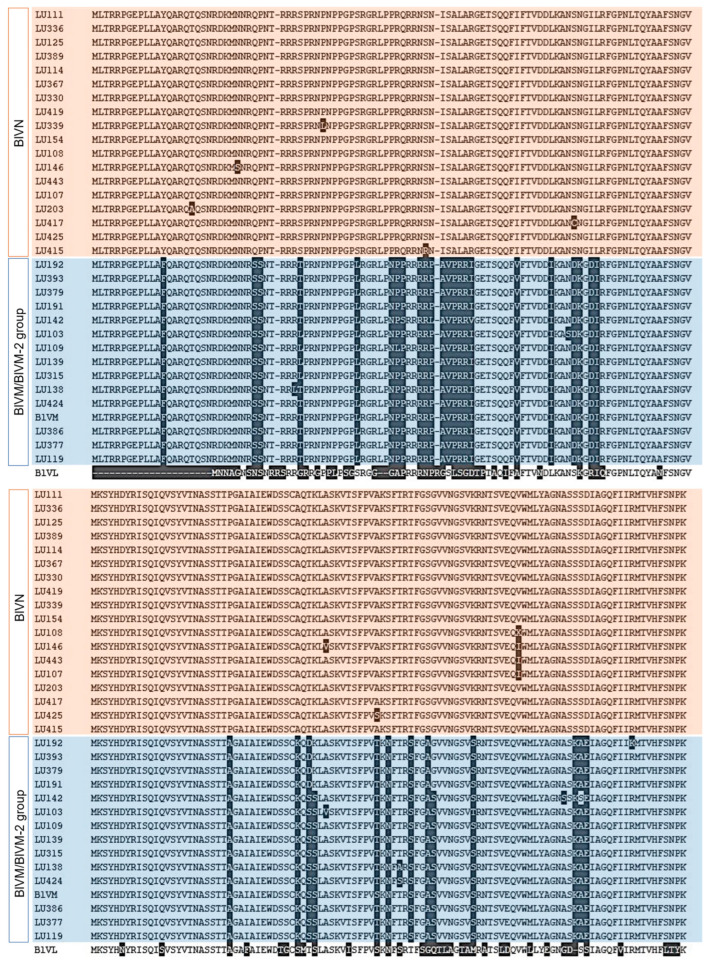
Amino acid sequence variation between full length translated coat protein (CP) of BlVN (highlighted in pink) and BlVM/M-2 (highlighted in blue) isolates from BC with comparison to CP of BlVM isolate, OR051501.1 and CP of BlVL isolate, OQ686746.1.

We amplified and sequenced the CP-encoding region to assess BlVN and BLVM/M-2 virus diversity in the Fraser Valley (Figure S3b). Dendrograms for both nucleotide and amino acid sequences showed that the 32 sequences separated into two groups, with 18 showing high similarity to BlVN (GenBank accession numbers PV893093-PV893110) and 14 showing high similarity to BlVM/M-2 (GenBank accession numbers PV935499-PV936512) consensus sequences (Figure S4). The full length MSA of translated CPs of 32 isolates is shown in Figure 2. CP of BlVN isolates contained more than 98% seqID in both nucleotide and amino acid levels and diverged from BlVM strains with 17% to 19% differences in seq ID, respectively. BlVM-like isolates were compared to the published BlVM sequence (OR051501.1). Among 14 isolates, 10 isolates contained 95–96.5% of nucleotide seqID and rest contained more than 99% nucleotide seqID. However, the amino acid sequence of CP in all BlVM-like isolates contained more than 97% identity. The CP sequence of BlVL (OQ686746.1) was 23 amino acids shorter than BlVN and BlVM/M-2 CP sequences and contained less than 60% similarity (Figure 2). We developed primers to differentiate BlVN and BlVM/M-2 using the variable regions in CP (Table 2). The primers were validated by testing 28 known positive samples for luteovirus (Figure S3c,d). Among them, 17 tested positive for BlVN and 21 were positive for BlVM/M-2, while 10 of the samples contained mixed infections of both BlVN and BlVM/M-2.

#### 3.4.2. Full-Length Genome Sequencing

De novo assembly of short reads can lead to artificial indels, especially if a mix of variants is present in the same sample [35]. To assess the overall sequences of BlVN and BlVM-2 genomes, we used long read sequencing of amplicons to achieve near full length genome sequences of seven BlVN isolates (GenBank accession numbers PX311070-PX311076) and eight BlVM isolates (GenBank accession numbers PX057975-PX057983). The maximum length of the sequence obtained was 4882 base pairs, with a read length N50 of 2755. The mean read quality was 11.9, with an average of 677 times coverage (Table S3). The 5′ and 3′ ends of BlVN were completed by Rapid Amplification of cDNA Ends (RACE) to generate the full-length genome sequence with 5089 bp. The BlVM-2 genome sequence was completed by both RACE and pair-wise alignment to the BlVM reference sequences [11]. Full-length genomes of BlVN isolate LU425 and BlVM-2 isolate LU387 from de novo assembly of Illumina reads were validated by Nanopore sequencing of full-length PCR products and submitted to GenBank under the accession numbers PX361093 and PX361092, respectively.

**Figure 3:**
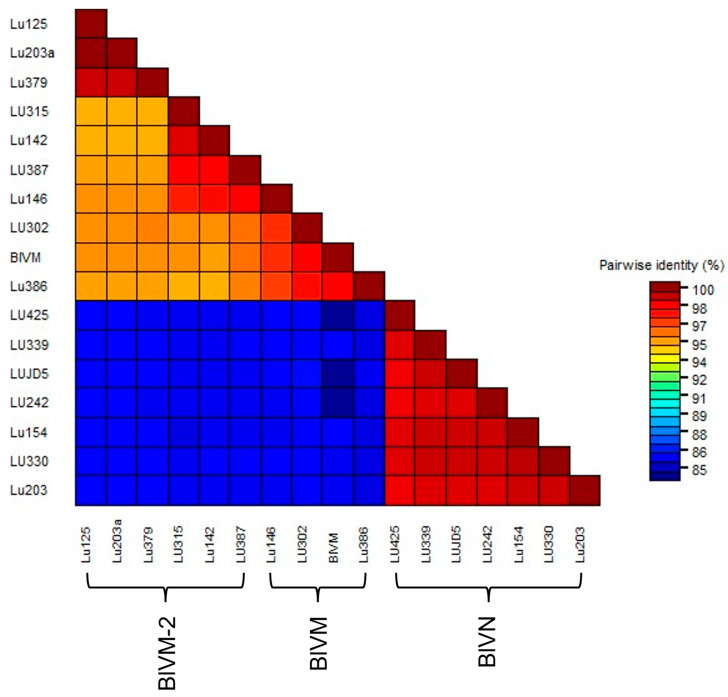
Pair-wise comparison between near full-length sequences of BlVN and BlVM/M-2 isolates collected from blueberry growing regions of Fraser Valley, BC, including Abbotsford (LU203, LU203a), Delta (LU125, LU146, LU142), Pitt Meadows (LU379, LU386, LU387, LUJD5), Langly (LU242), Chilliwack (LU154), Maple Ridge (LU339), and Surrey (LU302, LU315, LU330, LU425).

**Figure 4:**
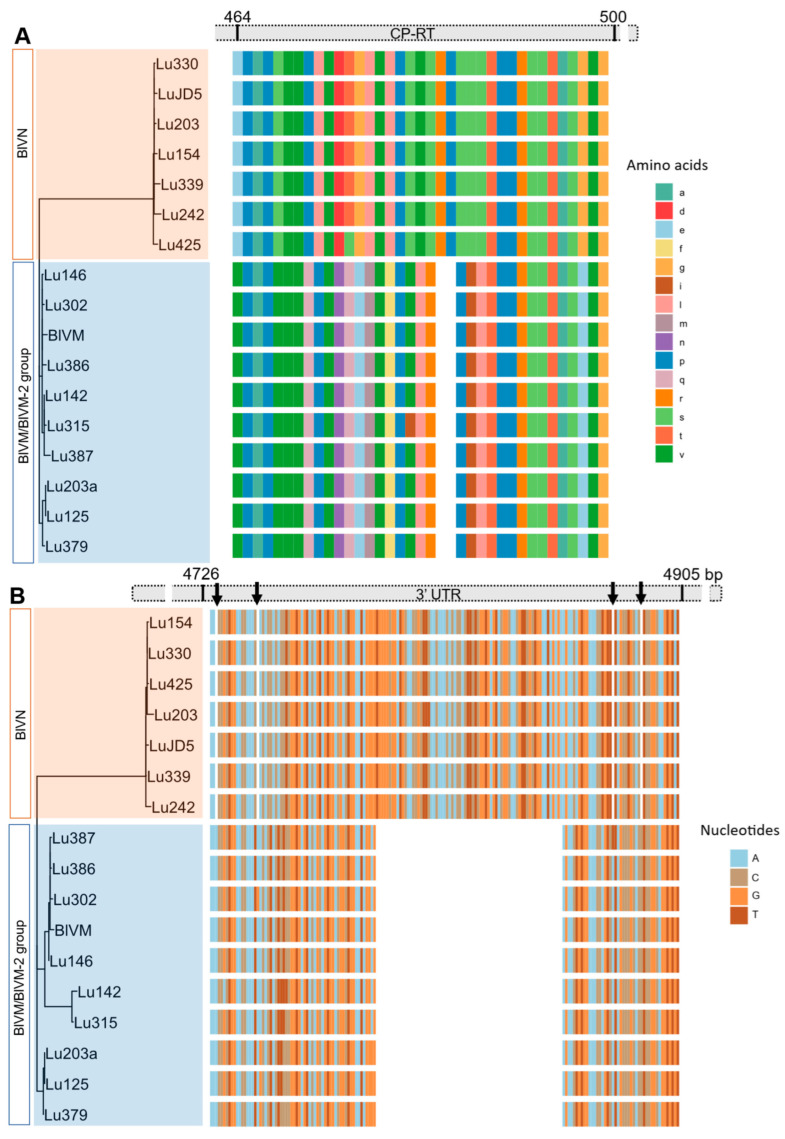
Indels in the blueberry virus M/M-2 genome sequence compared to blueberry virus N. (**A**) Alignment of amino acid sequences of coat protein with readthrough (CP-RT) showing the two amino acids (six base pairs) deletion in BlVM/M-2 sequences. (**B**) Alignment of nucleotide sequence of 3′UTR showing the 70-nucleotide deletion of BlVM/M-2 sequences and minor nucleotide deletions in BlVN (indicated with arrows).

The MSA of partial BlVN genome sequences showed 99% N-seq ID to each other and 83% N-seq ID to BlVM-like genome sequences. The MSA of BlVM-like partial genome sequences showed that three isolates were highly related to the previously reported BlVM genome sequence, OR051501.1, LU302, LU386 with the N-seq ID of 98%, and LU146 with the N-seq ID of 97% whereas the remaining isolates were more divergent with 95–96% N-seq ID to OR051501.1, confirming them as BlVM-2 variants (Figure 3). The BlVN and BLVM/BlVM-2 sequences were distinguished also by two consistent indels in the CP readthrough region and 3′ UTR region. The CP readthrough region of BlVN is two amino acids longer than that of BlVM/M-2, resulting from a six-nucleotide indel (Figure 4A). Moreover, both sides of this indel site showed amino acid sequence polymorphisms between BlVN and BlVM/M-2 sequences (Figure 4A). The second indel was in the 3′ UTR region. A 70-nucleotide deletion was located at the 3′ UTR of BlVM/M-2 and four minor deletions (one to two nucleotides) were located at the 3′ UTR of BlVN (Figure 4B). We used this 70 bp deletion to successfully differentiate BlVN and BlVM/M-2 using a duplex RT-PCR assay. This assay can readily be used for rapid detection of single or mixed infections (Table 2, Figure S3e).

## 4. Discussion

Here we report for the first time the presence of luteoviruses in highbush blueberry plants in Canada. We found them after sequencing rRNA-depleted RNA from diseased and healthy blueberry plants collected across the Fraser Valley of BC to identify potentially novel viruses. In addition to known disease-causing blueberry scorch and shock viruses, we identified four blueberry luteoviruses novel to blueberry plants in this region. Two of them were nearly identical to the BlVL and BlVM recently found in the USA [4,11]. The third genome is a major variant of BLVM, and we refer to it a BLVM-2. The fourth genome had 16% and 36% sequence differences when compared to the BlVM and BLVL genomes, respectively, identifying it as a novel blueberry luteovirus. We named it blueberry Virus N (BlVN). Here we will discuss similarities and differences between the predicted proteins of these genomes, as well as spread and potential role in disease.

Both BlVN and BlVM contained four open reading frames (ORF) and 5′ and 3′ untranslated regions. BlVN also contained a fifth putative ORF (see below). Based on sequence similarity to previously characterized blueberry luteoviruses and other luteoviruses [4,11,18], ORF1-2 encodes a putative P1-P2 protein with viral replicase activity. Similarly, the ORF3 of BlVN and BlVM/M2 encode putative coat proteins. ORF3 ended with an amber (UAG) stop codon, which is conserved in luteoviruses and known to promote a translational readthrough to express a C terminally extended minor coat protein readthrough domain (CP-RTD) [13,21]. Translated CP-RTD of BlVN contained 348 amino acids, whereas the BlVM contained 346 amino acids, due to the identified six nucleotide indel (Figure 4). This RTD domain is known to facilitate vector transmission of the virus [17,21]. Further, we found a potential ORF (ORF5b) embedded in the CD-RTD of BlVN isolates. Although BlVN also has an ORF5 embedded in ORF5 [4], the two embedded ORFs had only an average of 61% sequence similarity with 51% query coverage (Figure S4). The location of the embedded ORFs was also different. Both embedded ORFs have two predicted transmembrane helices (using TMHMM 2.0 software) [4]. Consistent with BlVL and BlVM [4], the BlVN and BlVM2 genomes lacked a fourth ORF that encodes a movement protein in other luteoviruses.

Luteoviruses generally do not have a 5′ cap structure or a poly-A tail. Instead, 5′ UTR and 3′ UTR secondary structures are important for their replication and translation [13,21]. We evaluated the sequences of the 5′ and 3′ UTRs of BlVN and BlVM2. The 5′ UTRs of BlVN and BlVM/M2 are 98% similar. Comparatively, 3′ UTRs had more variation, including a 70 base pair sequence missing in the BlVM/M2 3′UTR, and an overall 73% seqID. The function, if any, of the polymorphisms described here is currently unknown. The 70 base pair indel provides a simple diagnostic to separate BlVN from BlVM/M2 in plant samples based on amplicon length dimorphism when PCR primers flanking this sequence are used (Table 2).

Further screening by PCR showed that these luteoviruses are widespread in both healthy and diseased plants harbouring BlScV and BlShV in the Fraser Valley. Since we found BlVM-2 and BlVN in healthy plants, it is plausible that their effects on plant health may be subtle to none. However, the assessment was cursory and did not involve a comparison of plants without and with single or multiple luteovirus species under comparable conditions, precluding a conclusion about pathogenicity at this point. Similarly, since the diseased plants harboured BlScV or BlShV or both and displayed the wide range of symptoms caused by these viruses over time, we cannot make any inferences about a role of the luteoviruses in the disease development. Since combinations of viruses can result in both additive and synergistic effects [36,37], stock material for propagation should ideally be virus free. Together with the identification of BlVL and BlVM [4,11], the identification and sequence characterization of multiple isolates of BlVN and BLVM-2 as well as primers suitable for PCR testing in this study provide the information needed to identify luteovirus-free blueberry variety stocks before propagation and sale from nurseries.

## Figures and Tables

**Table 1:** Primers designed to amplify the coat protein encoding region of blueberry luteovirus.

Primer	Sequence	Target	Ta (°C)	Band Size (bp)
LutCDNA	GGTAGTCGAGCTGGCATTAGT	For cDNA synthesis		
LutF2	AATCCACCAGGGCCTTTAC	Detect BlVN and BlVM/M-2	57	238
LutR1	GTCGAGCTGGCATTAGTGA			
VcActin_C	CATCAAAGCATCGGTGAGATCC	For cDNA synthesis		
VcActin_F	AGGCTAACCGTGAGAAGATGAC	Control	57	128
VcActin_R	AGAGTCCAGCACGATTCCAG			
Lu3543 R	CCAAAAACGGGGAGAAGG	For cDNA synthesis		
Lu2598 F	CCCGTGGTGTAAAGAGATTG	Full coat protein amplification	63 *	850
Lu3447 R	CAACATTGAGCCTTTTCACG			

* Annealing temperature when using Q5^®^ High-Fidelity DNA Polymerase.

**Table 2:** PCR primers to differentiate between BlVN and BlVM/M-2 based on their sequence variations.

Target	Location	Primer	Sequence	Tm
BlVN	Coat protein CDS	Lu3103 F	TTGCCAAGAGCTTCACCAGGA	60
		Lu3202 R	TTGCCTGCGTACAACATCCA	
BlVM/M-2	Coat protein CDS	LuM2918 F	GCCAACGATAAAGGTGACATCCGGTT	64.7
		LuM3054 R	TCGAGCTGGCATTAGTCACGTACG	
BlVN and BlVM/M-2	3′UTR	Lu4472 F	AGTTCGAAACTCGGGGTTTGTCAAGC	63.4
		Lu5034 R	ACGATCGTAGATACTGCATCCCCA	

**Table 3:** Sequence identity values (%) to compare genomes and gene products of BlVN, BlVM-2, with BlVL (OQ686746.1) and BlVM (OR051501.1).

Blueberry Luteovirus	ORFs	% Nucleotide Sequence Similarity/Identity		
		Blueberry Virus L	Blueberry Virus M	Blueberry Virus M-2
Blueberry virus N	Genome	64.2	84.3	84.3
	ORF1	66.9	81.6	82.0
	ORF2	77.3	90.8	90.8
	ORF1-2	73.2	87.2	87.3
	ORF3 (CP)	62.4	82.0	82.3
	ORF3-5	62.4	84.0	84.0
	ORF5	62.4	85.2	85.0
Blueberry virus M-2	Genome	63.6	96.8	
	ORF1	66.4	93.4	
	ORF2	76.8	96.6	
	ORF1-2	72.7	95.3	
	ORF3 (CP)	61.7	96.8	
	ORF3-5	62.1	98.2	
		% amino acid sequence similarity		
	ORF1	66.7	87.7	88.3
Blueberry virus N	ORF1-2	77.6	92.8	93.0
	ORF3 (CP)	58.9	82.8	82.8
	ORF3-5	57.7	83.8	84.4
	ORF1	67.0	97.0	
Blueberry virus M-2	ORF1-2	76.8	98.5	
	ORF3 (CP)	60.5	98.6	
	ORF3-5	59.0	98.6	

**Table 4:** Percentage of blueberry luteovirus (BlVN/BlVM) positives in blueberry leaf samples collected from Fraser Valley, BC using RT-PCR assay.

Nearest City	Plant Type	Number of Plants Tested	Luteovirus Positives	% Positives
Abbotsford	Diseased	46	40	87
Aldergrove	Diseased	3	3	100
Chilliwack	Diseased	20	20	100
Delta	Diseased	39	35	89.7
Langley	Diseased	28	27	96.4
Maple Ridge	Diseased	7	6	85.7
Pitt Meadows	Diseased	40	34	85
Surrey	Diseased	30	29	96.7
Commercial fields	Healthy	14	7	50
Nursery varieties	Healthy	14	4	28.6

**Table 5:** Number of plants that tested positive for blueberry virus N/M and blueberry luteovirus L in symptomatic blueberry leaf samples collected from the Fraser Valley, and Vancouver Island, BC.

Nearest City	Number of Plants Tested	BlVN, BlVM/M-2 Positives	BlVL Positives
Abbotsford	64	60	63
Aldergrove	2	2	2
Chilliwack	32	29	30
Delta	8	6	8
Langley	10	10	9
Mission	6	5	5
Pitt Meadows	2	1	2
Vancouver Island	10	9	10
Surrey	19	16	17

## Data Availability

Data available in a publicly accessible repository: The data presented in this study are openly available in genbank as several accession numbers listed in the manuscript.

## Supplementary Materials

The following supporting information can be downloaded at: https://www.mdpi.com/article/10.3390/v17101286/s1, Table S1: Primer sequences used to amplify BlVN and BlVM genomes; Table S2: Percentage pair-wise sequence identity between blueberry Virus L (OQ686746.1) and de novo assemblies of BlVL-like isolates from BC (Clustal Omega, Geneious Prime 2023.2.1); Table S3: Long read sequencing for Luteovirus isolates; Figure S1: Alignment of translated amino acid sequences of partial P1-P2 protein of luteoviruses showing the conserved motifs (A) Partial P1-P2 protein sequence (266–532 aa) of blueberry luteovirus L, M, M-2 and N showing conserved motifs of helicase activity (motif III, IV and VI). (B) Partial P1-P2 protein sequence (478–723 aa) of blueberry luteovirus L, M, M-2 and N showing conserved motifs of RNA dependant RNA polymerase activity (motifs I to VIII). Green squares highlight the identical sequences and red squares shows variation of sequences between blueberry virus L, N and M/M-2; Figure S2: Phylogenetic relationship between blueberry luteoviruses and related luteoviruses. (A) phylogenetic tree constructed with amino acid sequences of P1-P2 fusion protein and (B) coat protein readthrough protein of blueberry luteovirus with other closely related luteovirus species (GenBank) using maximum likelihood method with the Le and Gascuel (LG) and Whelan and Goldman (WAG) models, respectively with 1000 bootstrap replications and, percentage consensus support indicated on each node (MEGA12); Figure S3: Gel image showing band sizes for different RT-PCR assays used to detect Blueberry luteovirus N and /M-2. (a) RT-PCR assay with LutF2 and LutR1 to detect presence of any BlVN and/or BlVM/M-2, (b) RT-PCR assay with Lu2598F and Lu3447R to amplify and sequence BlVN and BlVM/M-2 coat protein coding region, (c) RT-PCR assay with Lu3103F and Lu3202R to identify BlVN, (d) RT-PCR assay with LuM2918F and LuM3054R to identify BlVM/M-2, and (e) RT-PCR assay with duplex primers, Lu4472F and Lu5034R to differentiate BlVN and BlVM/M-2 in a single reaction. All PCR products were separated and visualized in 1.8% agarose gel except e, where 2% high resolution grade 3:1 agarose was used. Each gel was run with DNA ladder (L) and no template negative control (NC); Figure S4: Phylogenetic tree for translated amino acid sequence of coat protein of blueberry luteovirus isolates found in 32 diseased blueberry samples collected from Fraser Valley, BC, with maximum likelihood method and Jones-Taylor-Thornton (JTT) model with 1,000 bootstrap replications. Branches with more than 50% consensus support were shown in the tree. Isolates closely related to BK035257 are named as blueberry virus N, isolates closely related (≥98.5%) to blueberry virus M (OR051501.1) are labelled as blueberry virus M and isolates 97%-98.1% related to blueberry virus M are named as blueberry virus M-2; Figure S5: Comparison of putative ORF embedded in coat protein readthrough region. (A) A diagram showing the location of putative ORF5 in BlVN compared to BlVL. (B) The sequence identity between ORF5 translation of BlVN and putative P5b protein sequences of BlVL retrieved from NCBI.
