# Supplementary material for: Characterization of Novel Luteoviruses in Canadian Highbush Blueberries Using High-Throughput Sequencing
Source: Viruses. 2025 Sep 23;17(10):1286. doi: 10.3390/v17101286 (PMC12568157; doi:10.3390/v17101286)
Supplement: Supplementary file 1 [file viruses-17-01286-s001.zip › viruses-3854370-supplementary.docx]

**Supplementary File**

**Characterization of Novel Luteoviruses in Canadian Highbush Blueberries Using High-throughput Sequencing**

Sachithrani Kannangara^1^, Adam Gilewski^1^, Juan Rodriguez Lopez^1^, Gertruida de Villiers^2^, Meghan Ellis^2^, Peter Ellis^2^, Eric Gerbrandt^3^, Jim Mattsson^1*^

^1^Simon Fraser University, Department of Biological Sciences, 8888 University Dr, Burnaby BC V5A1S6, Canada

^2^Phyto Diagnostics Company Ltd., 9381 Ardmore Dr, North Saanich BC V8L 5G4, Canada

^3^British Columbia Blueberry Council 32160 S Fraser Way, Abbotsford BC V2T 1W5, Canada

**Table S1** Primer sequences used to amplify BlVN and BlVM genomes

| Primer name | Sequence | Usage |
| --- | --- | --- |
| LutL5,074 R | TCCCTGGGTCCCTATAAACATGGA | cDNA synthesis |
| LutL55 F | TGTAAGTCTTGCAAGCCGAACTGA | Long PCR |
| LutL5,074 R | TCCCTGGGTCCCTATAAACATGGA |  |
| LutL138 F | CCTCTGCCAAGGTTGTCAAGGATT | Nested PCR |
| LutL5,074 R | TCCCTGGGTCCCTATAAACATGGA |  |

**Table S2** Percentage pair-wise sequence identity between Blueberry Virus L (OQ686746.1) and de novo assemblies of BlVL-like isolates from BC (Clustal Omega, Geneious Prime 2023.2.1)

|  | Blueberry virus L | J20NODE_300_length_4993_cov_61.625102 | 25NODE_131_length_5048_cov_43.614180 (reversed) | S3NODE_159_length_5027_cov_75.955022 (reversed) | 7NODE_125_length_5028_cov_101.975600 | E13NODE_96_length_4998_cov_52.566646 | S1NODE_157_length_5020_cov_16.825692 |
| --- | --- | --- | --- | --- | --- | --- | --- |
| **Blueberry virus L** |  | **93.2** | **93.4** | **93.1** | **93.2** | **93.2** | **93.3** |
| J20NODE_300_length_4993_cov_61.625102 | 93.2 |  | 96.6 | 97.5 | 95.5 | 96.7 | 96.5 |
| 25NODE_131_length_5048_cov_43.614180 (reversed) | 93.4 | 96.6 |  | 98 | 96.4 | 96.1 | 97.2 |
| S3NODE_159_length_5027_cov_75.955022 (reversed) | 93.1 | 97.5 | 98 |  | 95.6 | 96.8 | 97 |
| 7NODE_125_length_5028_cov_101.975600 | 93.2 | 95.5 | 96.4 | 95.6 |  | 97.4 | 97.2 |
| E13NODE_96_length_4998_cov_52.566646 | 93.2 | 96.7 | 96.1 | 96.8 | 97.4 |  | 97.4 |
| S1NODE_157_length_5020_cov_16.825692 | 93.3 | 96.5 | 97.2 | 97 | 97.2 | 97.4 |  |

**Table S3** Long read sequencing for Luteovirus isolates

| Luteovirus | Isolate | Mean read quality | Total bases | Length of final contig | Mean coverage |
| --- | --- | --- | --- | --- | --- |
| BlVN | Lu425 | 12.3 | 2,791,098 | 4870 | 573.1 |
|  | Lu339 | 12.3 | 3,615,265 | 4868 | 742.7 |
|  | Lu203 | 11.6 | 2,744,786 | 4874 | 563.1 |
|  | LuJD5 | 12.7 | 3,490,515 | 4873 | 716.3 |
|  | Lu154 | 12.7 | 4,968,369 | 4873 | 1019.6 |
|  | Lu330 | 12.3 | 2,779,879 | 4871 | 570.7 |
|  | Lu242 | 12.4 | 3,729,294 | 4871 | 765.6 |
| BlVM-2 | Lu125 | 10.7 | 1,596,060 | 4799 | 332.6 |
|  | Lu203a | 10.6 | 2,550,341 | 4882 | 522.4 |
|  | Lu379 | 10.6 | 2,524,433 | 4799 | 526.0 |
|  | Lu315 | 12.2 | 3,709,519 | 4801 | 772.7 |
|  | Lu142 | 12.2 | 4,159,071 | 4796 | 867.2 |
|  | Lu387 | 12.5 | 4,152,854 | 4799 | 865.4 |
| BlVM | Lu146 | 12.6 | 3,724,303 | 4799 | 776.1 |
|  | Lu302 | 12.7 | 3,566,118 | 4799 | 743.1 |
|  | Lu386 | 10.9 | 2,288,666 | 4798 | 477.0 |


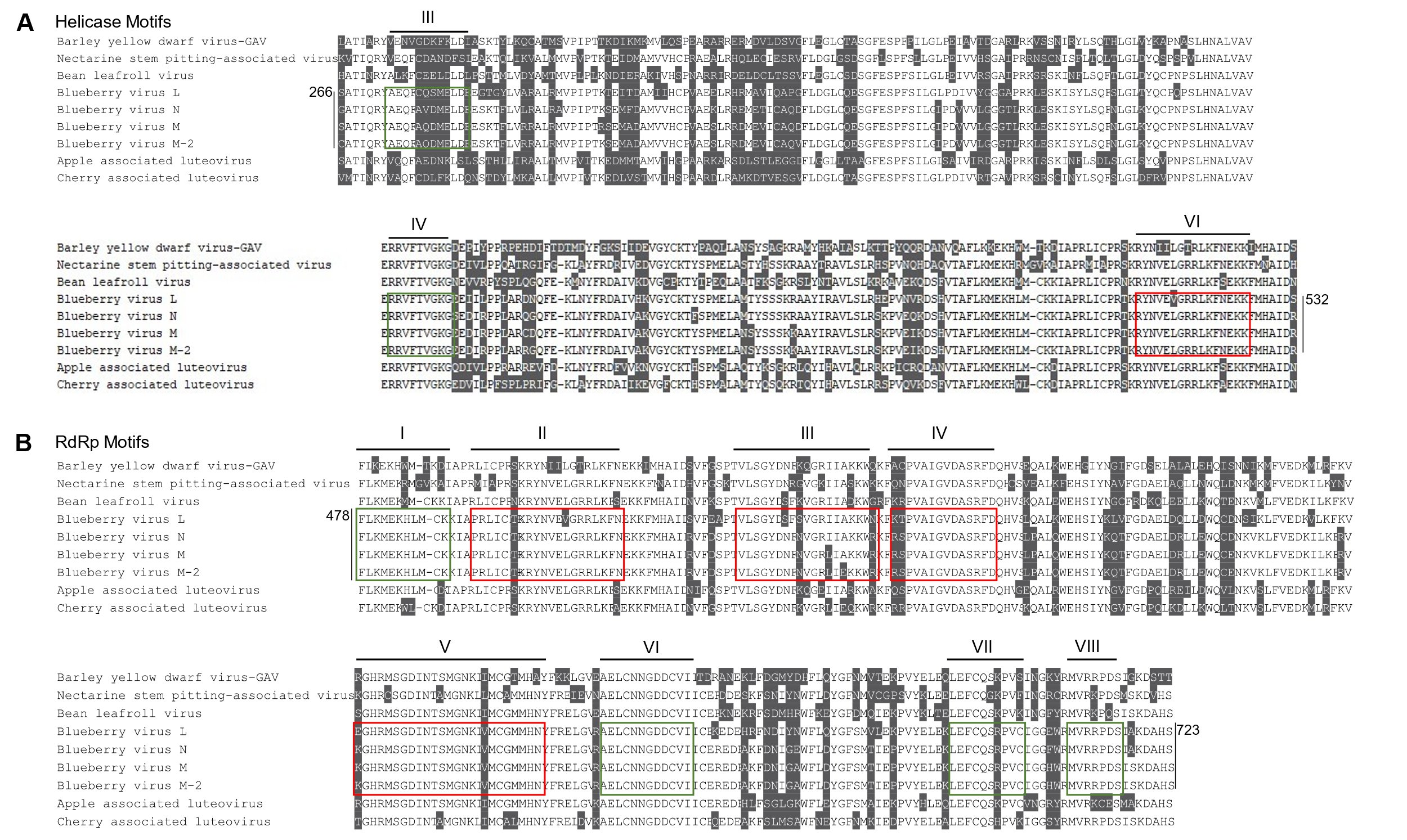


**Figure S1** Alignment of translated amino acid sequences of partial P1-P2 protein of luteoviruses showing the conserved motifs (A) Partial P1-P2 protein sequence (266-532 aa) of blueberry luteovirus L, M, M-2 and N showing conserved motifs of helicase activity (motif III, IV and VI). (B) Partial P1-P2 protein sequence (478-723 aa) of blueberry luteovirus L, M, M-2 and N showing conserved motifs of RNA dependant RNA polymerase activity (motifs I to VIII). Green squares highlight the identical sequences and red squares shows variation of sequences between blueberry virus L, N and M/M-2.


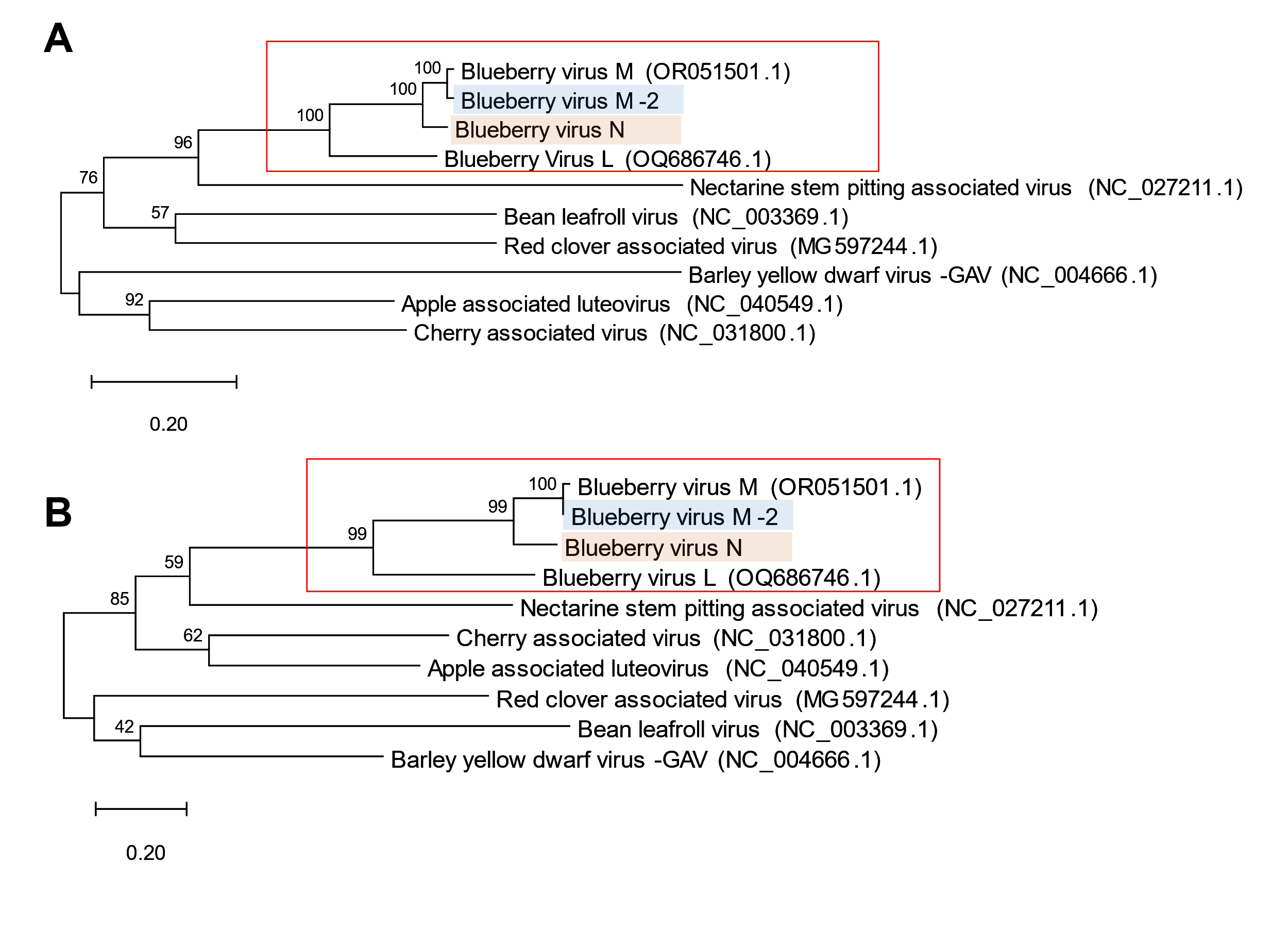
**Figure S2** Phylogenetic relationship between blueberry luteoviruses and related luteoviruses. (A) phylogenetic tree constructed with amino acid sequences of P1-P2 fusion protein and (B) coat protein readthrough protein of blueberry luteovirus with other closely related luteovirus species (GenBank) using maximum likelihood method with the Le and Gascuel (LG) and Whelan and Goldman (WAG) models, respectively with 1000 bootstrap replications and, percentage consensus support indicated on each node (MEGA12).

**
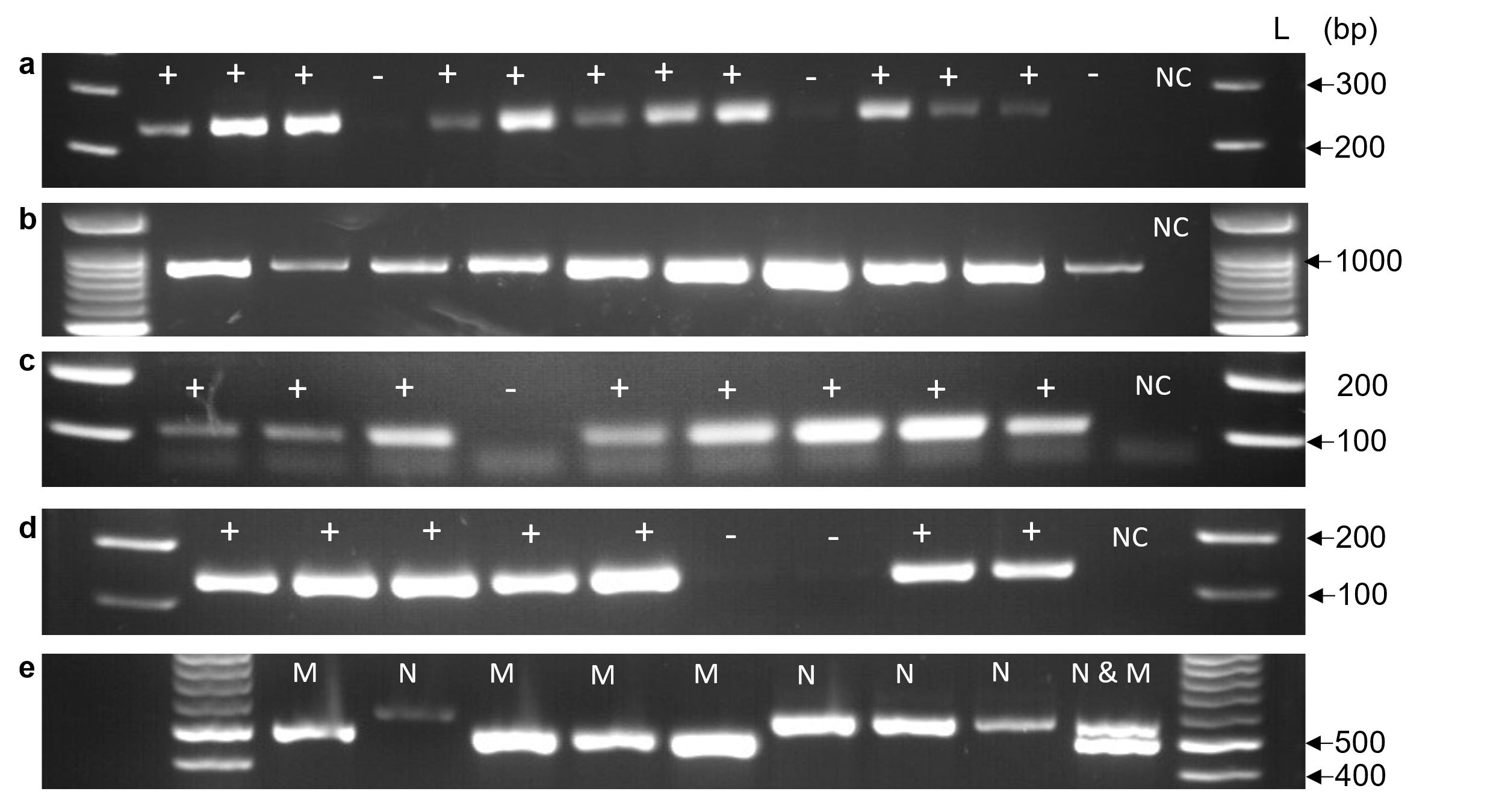
Figure S3** Gel image showing band sizes for different RT-PCR assays used to detect Blueberry luteovirus N and /M-2. (a) RT-PCR assay with LutF2 and LutR1 to detect presence of any BlVN and/or BlVM/M-2, (b) RT-PCR assay with Lu2598F and Lu3447R to amplify and sequence BlVN and BlVM/M-2 coat protein coding region, (c) RT-PCR assay with Lu3103F and Lu3202R to identify BlVN, (d) RT-PCR assay with LuM2918F and LuM3054R to identify BlVM/M-2, and (e) RT-PCR assay with duplex primers, Lu4472F and Lu5034R to differentiate BlVN and BlVM/M-2 in a single reaction. All PCR products were separated and visualized in 1.8% agarose gel except e, where 2% high resolution grade 3:1 agarose was used. Each gel was run with DNA ladder (L) and no template negative control (NC).

**
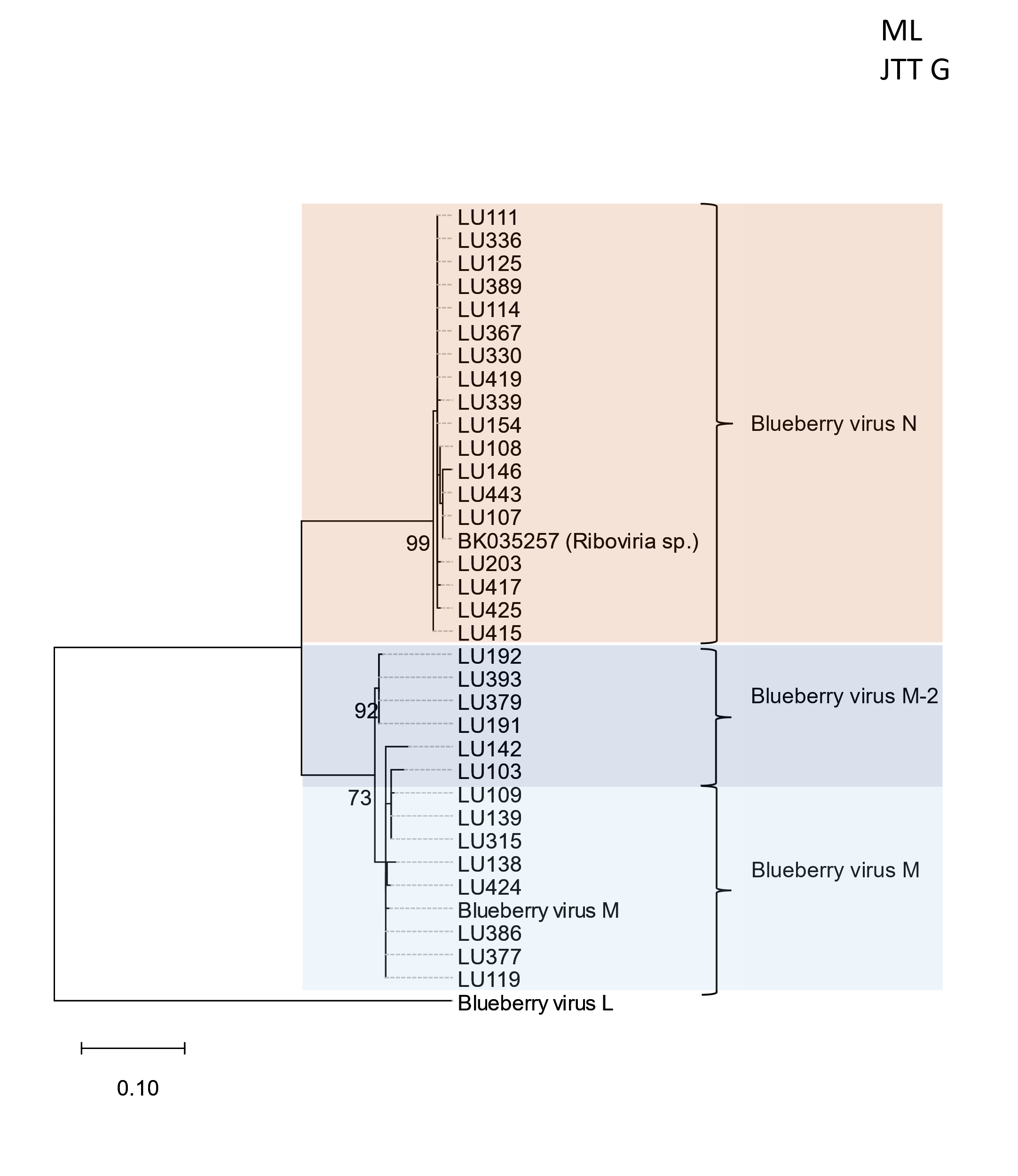
Figure S4** Phylogenetic tree for translated amino acid sequence of coat protein of blueberry luteovirus isolates found in 32 diseased blueberry samples collected from Fraser Valley, BC, with maximum likelihood method and Jones-Taylor-Thornton (JTT) model with 1,000 bootstrap replications. Branches with more than 50% consensus support were shown in the tree. Isolates closely related to BK035257 are named as blueberry virus N, isolates closely related (≥ 98.5 %) to blueberry virus M (OR051501.1) are labelled as blueberry virus M and isolates 97%-98.1% related to blueberry virus M are named as blueberry virus M-2.


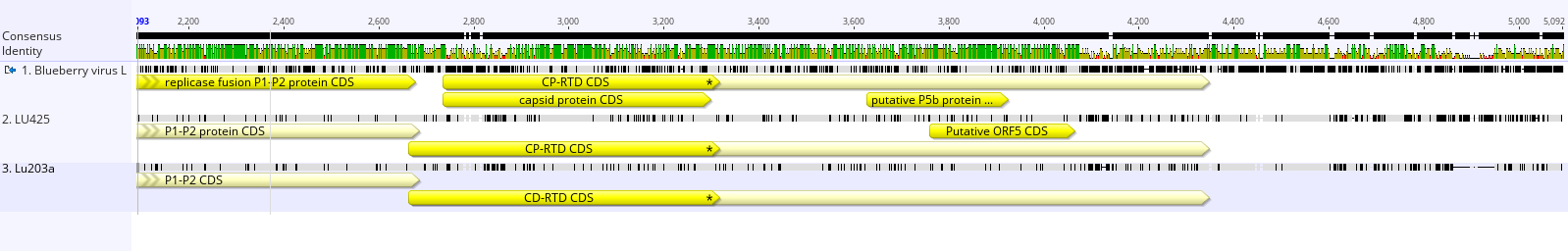

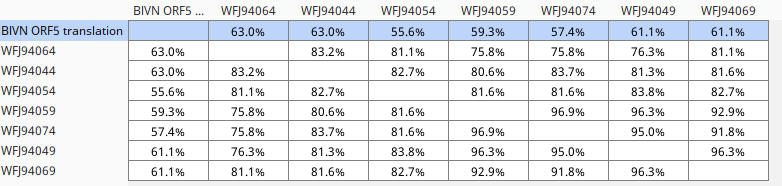


BlVL

BlVN

BlVM-2

**A**

**B**

**Figure S5** Comparison of putative ORF embedded in coat protein readthrough region. (A) A diagram showing the location of putative ORF5 in BlVN compared to BlVL. (B) The sequence identity between ORF5 translation of BlVN and putative P5b protein sequences of BlVL retrieved from NCBI.
